# The long term effect of pulmonary tuberculosis on income and employment in a low income, urban setting

**DOI:** 10.1136/thoraxjnl-2020-215338

**Published:** 2020-12-18

**Authors:** Jamilah Meghji, Stefanie Gregorius, Jason Madan, Fatima Chitimbe, Rachael Thomson, Jamie Rylance, Ndaziona PK Banda, Stephen B Gordon, Elizabeth L Corbett, Kevin Mortimer, Stephen Bertel Squire

**Affiliations:** 1 Department of Clinical Sciences, Liverpool School of Tropical Medicine, Liverpool, UK; 2 Malawi-Liverpool-Wellcome Trust Clinical Research Programme, Blantyre, Malawi; 3 Deutsche Gesellschaft fur Internationale Zusammenarbeit, Bonn, Germany; 4 Warwick Clinical Trials Unit, University of Warwick, Coventry, UK; 5 Department of Medicine, Queen Elizabeth Central Hospital, Blantyre, Malawi; 6 Department of Infectious and Tropical Diseases, London School of Hygiene and Tropical Medicine, London, UK

**Keywords:** Pulmonary tuberculosis, TB sequelae, post-TB lung disease, health economics, social determinants

## Abstract

**Background:**

Mitigating the socioeconomic impact of tuberculosis (TB) is key to the WHO End TB Strategy. However, little known about socioeconomic well-being beyond TB-treatment completion. In this mixed-methods study, we describe socioeconomic outcomes after TB-disease in urban Blantyre, Malawi, and explore pathways and barriers to financial recovery.

**Methods:**

Adults ≥15 years successfully completing treatment for a first episode of pulmonary TB under the National TB Control Programme were prospectively followed up for 12 months. Socioeconomic, income, occupation, health seeking and cost data were collected. Determinants and impacts of ongoing financial hardship were explored through illness narrative interviews with purposively selected participants.

**Results:**

405 participants were recruited from February 2016 to April 2017. Median age was 35 years (IQR: 28–41), 67.9% (275/405) were male, and 60.6% (244/405) were HIV-positive. Employment and incomes were lowest at TB-treatment completion, with limited recovery in the following year: fewer people were in paid work (63.0% (232/368) vs 72.4% (293/405), p=0.006), median incomes were lower (US$44.13 (IQR: US$0–US$106.15) vs US$72.20 (IQR: US$26.71–US$173.29), p<0.001), and more patients were living in poverty (earning <US$1.90/day: 57.7% (211/366) vs 41.6% (166/399), p<0.001) 1 year after TB-treatment completion compared with before TB-disease onset. Half of the participants (50.5%, 184/368) reported ongoing dissaving (use of savings, selling assets, borrowing money) and 9.5% (35/368) reported school interruptions in the year after TB-treatment completion. Twenty-one participants completed in-depth interviews. Reported barriers to economic recovery included financial insecurity, challenges rebuilding business relationships, residual physical morbidity and stigma.

**Conclusions:**

TB-affected households remain economically vulnerable even after TB-treatment completion, with limited recovery in income and employment, persistent financial strain requiring dissaving, and ongoing school interruptions. Measures of the economic impact of TB disease should include the post-TB period. Interventions to protect the long-term health and livelihoods of TB survivors must be explored.

**Key messages**

**What is the key question?**

- What is the lasting socioeconomic impact of tuberculosis (TB) disease on patients and households: can we assume full economic recovery after successful treatment completion, and what are the pathways and barriers to this recovery?

**What is the bottom line?**

- Many TB-affected households experience a limited recovery in income and employment in the year after TB-treatment completion, with ongoing dissaving and school interruptions. Barriers to economic recovery include persistent financial insecurity, challenges rebuilding business relationships, residual physical morbidity and stigma.

**Why read on?**

- The socioeconomic impact of TB disease persists well beyond TB-treatment completion. Understanding this process, and developing strategies to mitigate this, will be crucial if we are to meet the WHO End TB Strategy target of eliminating TB-related catastrophic costs by 2030, and improve the long-term well-being of TB survivors.

## Introduction

An estimated 10 million incident cases of tuberculosis (TB) disease occurred globally in 2018, one-quarter of which occurred in Africa where 29% of patients are HIV coinfected.^1^ The early costs associated with TB disease in low-income settings are well recognised: despite provision of free TB-treatment services within the public sector, patients incur direct costs related to health seeking, indirect costs from lost income, and dissaving (the use of savings, borrowing of money, or sale of household assets) over the course of illness, diagnosis and treatment.^2 3^ These early costs are widespread, frequently profound and have been associated with adverse TB-treatment outcomes including treatment failure, loss to follow-up or death.^4^ However, surprisingly little is known about the lasting economic impact of disease beyond TB-treatment completion, and facilitators or barriers to economic recovery.

The physical effects of pulmonary-TB (PTB) are felt long after treatment completion: mortality rates are three to fourfold higher among TB survivors compared with TB-naïve adults,^5^ TB survivors have a high risk of disease recurrence,^6^ and the burden of residual post-TB lung disease (PTLD) is marked.^7 8^ Limited data are available on long-term psychosocial morbidities, but reports from TB-affected communities suggest these are considerable.^9 10^ It is plausible that the ongoing physical and psychosocial impact of TB disease is accompanied by long-term economic harm. Understanding this link will be key to improving the overall well-being of TB survivors,^11 12^ and essential if we are to meet the WHO End TB Strategy target of eliminating catastrophic costs for all TB-affected households by 2030.^13^

Malawi is one of the poorest countries in the world,^14^ with an estimated national TB incidence of 181/100 000 in 2018.^15^ In this mixed-methods study, nested within a prospective cohort of adults successfully treated for PTB in urban Blantyre, Malawi,^16^ we describe economic morbidity in the year after TB-treatment completion, and explore its determinants and impacts.

## Methods

Full methods of the parent cohort study have been described previously.^16^ In brief, 405 sequential HIV-positive and HIV-negative patients successfully completing treatment for PTB were recruited between February 2016 and April 2017 in urban Blantyre, Malawi. Inclusion criteria were age ≥15 years, and successful completion of treatment for a first episode of TB as defined by the National TB Control Programme (NTP). We excluded patients who had been treated for multidrug resistant disease. All participants provided written informed consent.

Study visits were conducted within 1 month of TB-treatment completion, and at 6 and 12 months thereafter. Questionnaires were completed in the local language, Chichewa. We collected data on demographics, socioeconomic situation (SES), TB and respiratory symptoms, quality of life, main occupation and income at each study visit. Patients provided information on occupation and income prior to TB illness from memory. Data on ongoing health seeking and associated direct and indirect costs were collected prospectively. Occupation was described using categories defined by the Malawi Demographic Health Survey 2015–2016.^17^ Income and dissaving questions were adapted from the STOP TB costing questionnaire.^18^ Monthly income was defined as money received by the individual, from work or other means, and was standardised into US dollars (online supplemental S1 appendix). Socioeconomic status was defined at TB-treatment completion using the Malawi EquityTool 2012.^19^ Information on TB microbiology at diagnosis was collected from NTP registers. HIV care is provided in a separate programme in this setting, and patient-held health passports were therefore reviewed to ascertain HIV status and antiretroviral therapy (ART) use, with HIV testing offered to all those with unknown serostatus who had not had a documented test within the past 1 month (Serial testing with Determine 1/2; Alere, USA/Uni-Gold; Recombigen HIV, Trinity Biotech, Ireland). CD4 counts were measured in all HIV-positive participants.

10.1136/thoraxjnl-2020-215338.supp1 Supplementary data

Illness narrative interviews were conducted with purposively selected patients who had completed TB treatment ≥12 months previously, in order to explore their experiences of TB illness and recovery.^20^ Recruitment ensured variation in gender, HIV status and socioeconomic status, and was stopped at the point of saturation. Interviews were conducted in Chichewa by a Malawian research assistant in a private location of the participant’s choice, most frequently their home, using a predesigned interview guide structured around the illness trajectory (life before, during and after TB treatment), which addressed issues of health, healthcare seeking behaviour and experiences, socioeconomic well-being, family and community life (online supplemental S2 appendix). Interviews were audiorecorded, transcribed into Chichewa, and translated into English. Notes and observations recorded by the study team were included as primary data.

All participants were compensated for their time and travel costs, in keeping with local ethics guidelines. The amount received over the 1-year study period was the equivalent of US$15.30 per participant.

### Sample size

The sample size of 400 was predetermined by the parent study, in order to allow the prevalence of PTLD to be estimated with ±5% precision and 95% confidence.

### Data analysis

Quantitative data were analysed using Stata V.15 (StataCorp). Health economic parameters are presented for each time point using median (IQR) values. χ^2^ test, Fisher’s exact or Wilcoxon rank-sum tests were used for comparisons between participant groups, and McNemar’s test used for within-group comparisons over time. Individuals were classified by occupation into those with paid work (self-employed, formally employed (in government, non-governmental organisations, private sector), farming, domestic work, informal piece-work), unpaid work (housework, students) and no work (retired, unemployed). Participants were considered to be living in poverty if earning <US$1.90/day.^21^

Logistic and linear regression models were used to explore the relationship between PTLD and economic outcomes, controlling for prespecified covariates recorded at TB-treatment completion (age, gender, HIV status, TB microbiology, educational level). Qualitative data were analysed thematically using an inductive framework approach^22^: transcripts were discussed, a coding and thematic framework was developed iteratively, relationships between codes and themes were identified manually, and emerging links were cross-checked by discussion with the study team and triangulation with study team notes.

## Results

### Patient population

**Figure 1:**
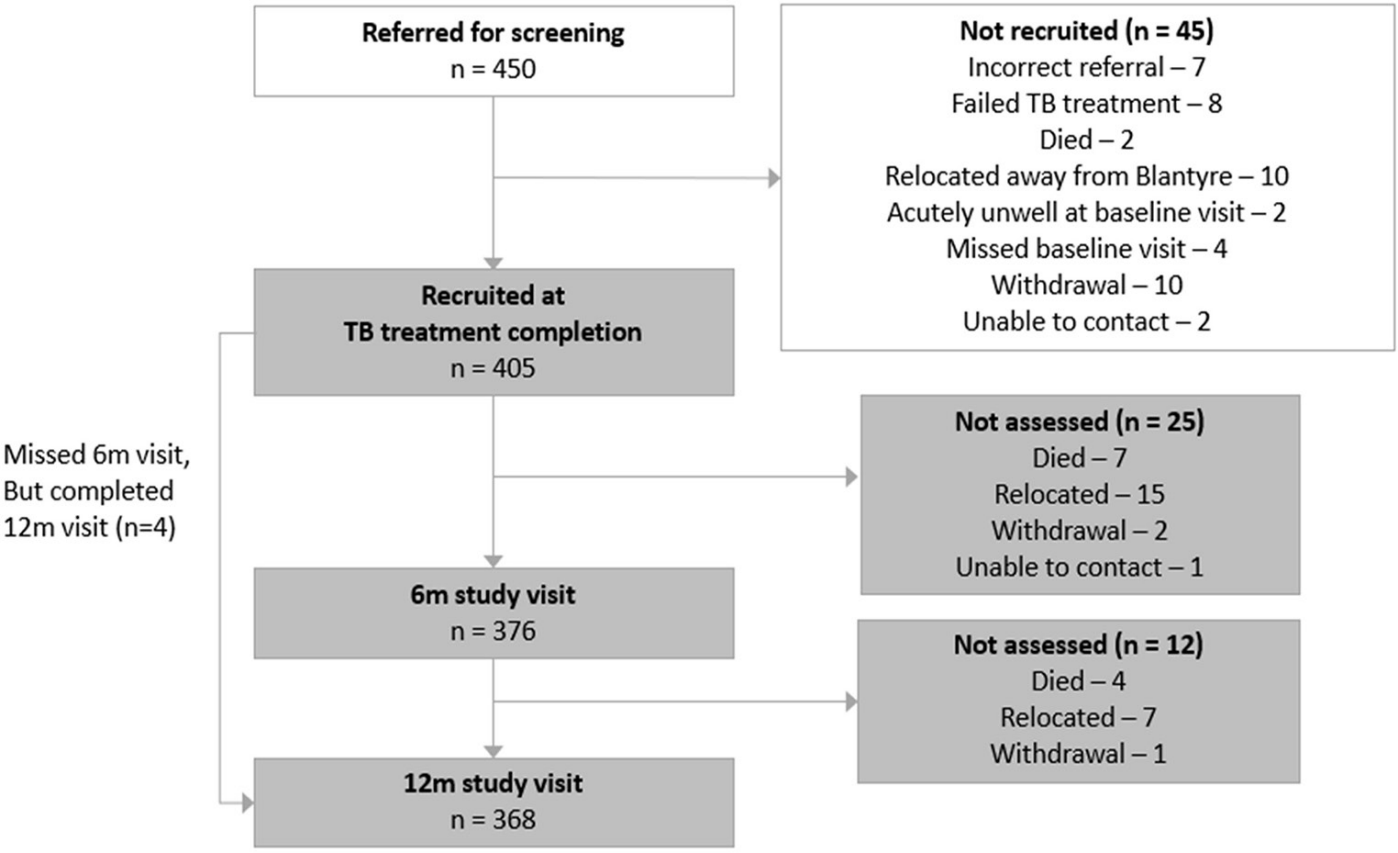
Participant flow diagram. TB, tuberculosis.

A total of 450 PTB survivors were screened, and 405 met inclusion criteria (figure 1). The 37/405 (9.1%) participants who did not complete the final study visit had similar characteristics (age, sex, HIV status, TB microbiology, SES) to those who completed the study, but lower average CD4 counts at TB-treatment completion (113 cells/µL (IQR: 62–197) vs 244 cells/µL (IQR: 137–398), p=0.007)(online supplemental S3 appendix).

The majority of participants (67.9% (275/405)) were male, 77.3% (313/405) had microbiologically proven PTB disease, and 60.6% (244/403) were HIV-positive. Over half were from the lowest three urban wealth quintiles (54.3% (202/372)) and 38.0% (154/405) had not attended school beyond primary level. The 21 participants purposively selected for in-depth interviews had similar demographic and socioeconomic characteristics to other study participants, but a longer duration of illness prior to TB treatment (13.0 vs 8.7 weeks, p<0.001), and less formal education (38.1 (8/21) vs 63.3% (243/384) beyond primary school, p=0.021) (table 1).

**Table 1:** Participant characteristics for participants included/ not included in nested qualitative work

Participant characteristic	Parent study only (n=384)	Parent and qualitative study (n=21)	p-value*
Age (years) (median, IQR)	34 (28–41)	35 (32–41)	0.246
Male sex (n, %)	261 (68.0%)	14 (66.7%)	0.901
Positive TB microbiology† at diagnosis (n, %)	299 (77.9%)	14 (66.7%)	0.233
Self-reported illness duration prior to TB treatment (weeks) (median, IQR)	8.7 (4.3–13.0)	13.0 (13.0–52.2)	<0.001
HIV-infected at TB-treatment completion (n=403)‡	232 (60.7)	12 (57.1)	0.743
ART use at TB-treatment completion (n, %) (n=244)	215 (92.7)	9 (75.0)	0.030
CD4 if HIV-positive at TB-treatment completion (cells/µL) (median, IQR) (n=242)	229 (127-397)	214 (126–420)	0.941
Maximum education level >primary school (n, %)	243 (63.3%)	8 (38.1%)	0.021
Urban SES quintile (n, %) (n=372) § Poorest Second poorest Middle Second most wealthy Most wealthy	21 (6.0%) 79 (22.5%) 87 (24.8%) 111 (31.6%) 53 (15.1%)	1 (4.8%) 6 (28.6%) 8 (38.1%) 3 (14.3%) 3 (14.3%)	0.449

*χ^2^ test for categorical variables, Wilcoxon rank-sum test for continuous variables.

†Microbiology positive if smear, culture or Xpert MTB/RIF positive.

‡HIV status missing for two study participants.

§ Urban household wealth quintiles calculated from household characteristic and asset data, using a tool validated by the Malawi Malaria Indicator Survey 2012 (Equity measurement Toolkit; Social Franchising Metrics Working Group)^19^

ART, antiretroviral treatment; SES, socioeconomic situation; TB, tuberculosis.

### Economic morbidity, after TB-treatment completion

#### Occupation and income

**Figure 2:**
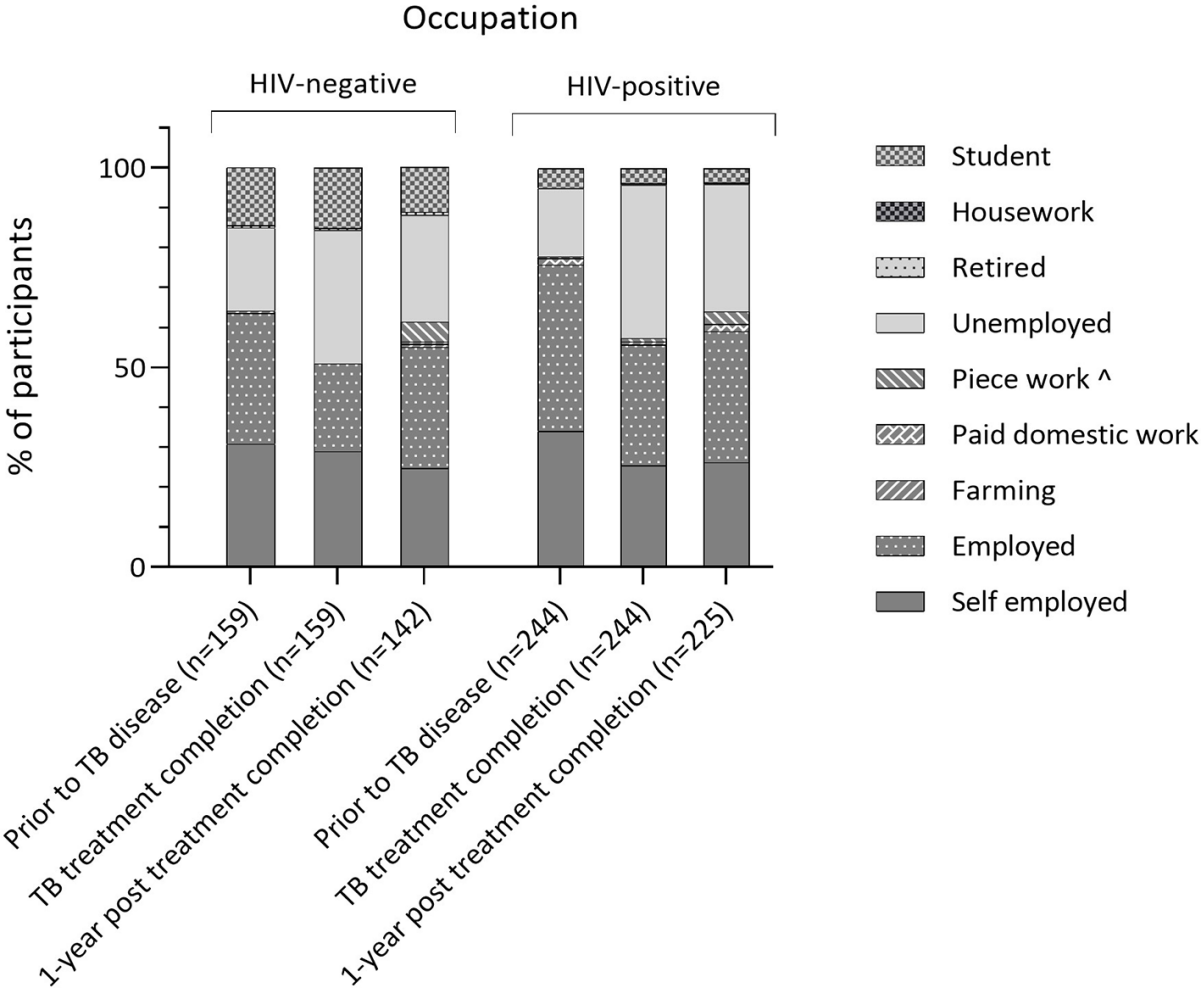
Self-reported occupation over time (prior to TB disease, at TB-treatment completion and 1-year after TB-treatment completion) stratified by HIV status.*HIV status available for 403 participants; ˆAd hoc work (eg, washing clothes, driving of minibuses, carrying goods for others). TB, tuberculosis.

The proportion of participants in paid work fell during TB disease to a nadir of 54.8% (222/405) at TB-treatment completion, with 36.5% (148/405) unemployment (National unemployment rates 5.4%–5.7% between 2016 and 2018).^23^ One year later, fewer participants were in paid work than prior to disease (63.0% (232/368) vs 72.4% (293/405), p=0.003), and the proportion of self-employed business people had not returned to previous levels (preillness: 32.8% (133/405); TB treatment end 26.9% (109/405); 1-year post-treatment completion 25.8% (95/368), p=0.026). Patterns were similar for HIV-positive and negative participants (figure 2).

A fifth of participants (20.7%, 74/358) moved from paid or unpaid work prior to TB disease, to no work by 1 year after TB-treatment completion. Half of these individuals lost work during TB disease and treatment (47.3%, 35/74), and half lost work in the year after treatment completion (52.7%, 39/74). Loss of work was more common in the lowest two versus highest three socioeconomic quintiles (28.6% (30/105) vs 15.9% (39/245), p=0.004). Among those who were employed prior to disease, 11.0% (17/154) had become self-employed by the end of follow-up.

**Figure 3:**
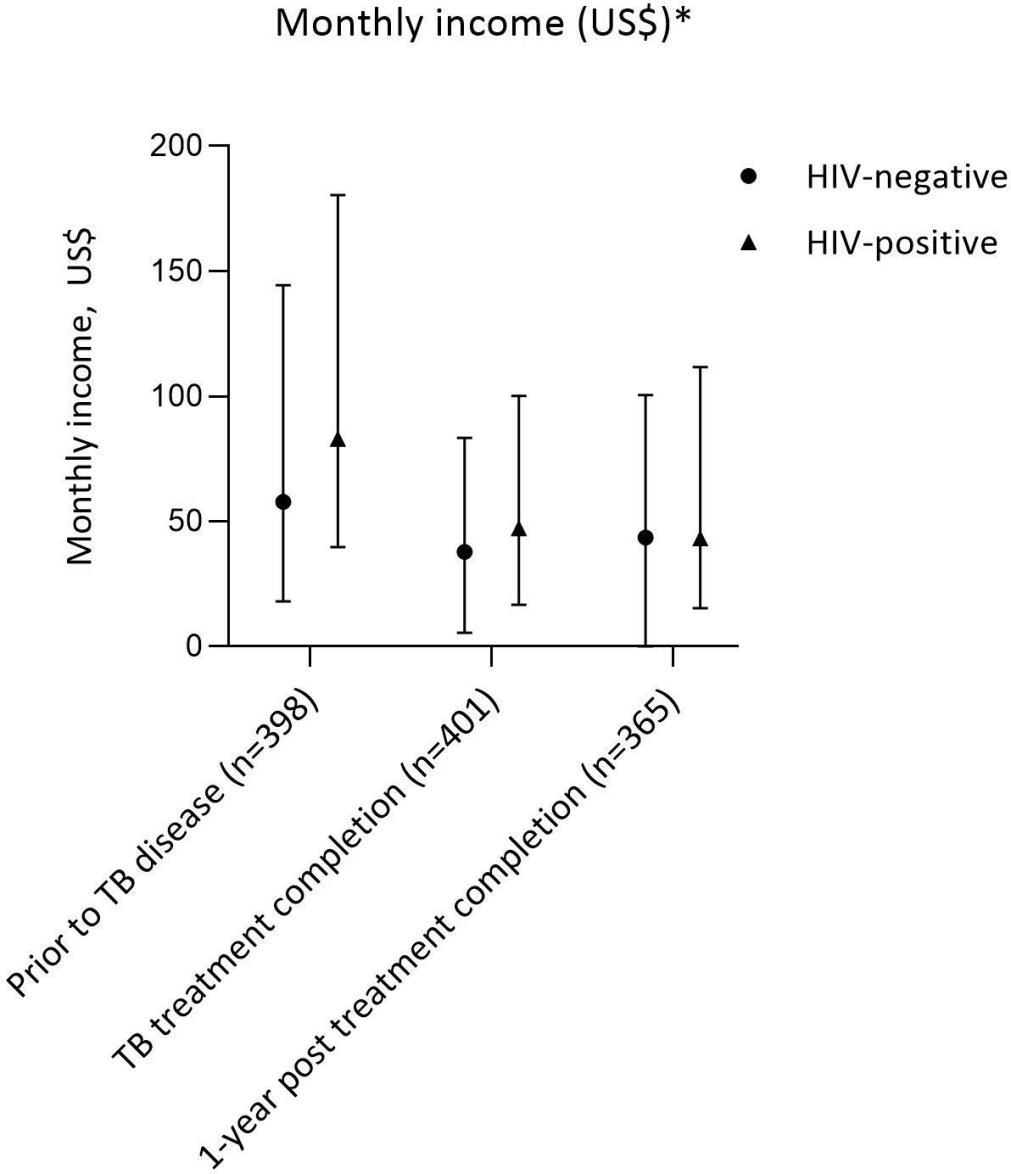
Self-reported income over time (prior to TB disease, at TB-treatment completion and 1-year after TB-treatment completion) stratified by HIV status (median, IQR)^#^.*Income data recorded in Malawian Kwacha with standardisation into US dollars by exchange rate at midpoint of pre-TB, baseline, and 1-year data collection intervals (online supplemental S1 appendix). ^#^HIV status available for 403 participants. TB, tuberculosis

Many participants did not know their total household income, including 40.7% (53/130) of women. However, individual incomes followed a similar pattern to that seen with work: median income was highest prior to TB illness, falling to a nadir at TB-treatment completion, with minimal recovery in the following year (figure 3). Monthly incomes fell by a median of $11.59 (IQR for income difference: −72.20 to +12.89) from pre-illness to 1-year post treatment completion, with the greatest loss among those who were originally self-employed (−US$74.96 (IQR: −231.99 to −7.01)). There was little difference in the median income loss experienced by the poorest two and wealthiest three socioeconomic quintiles over this period (−US$11.78 (IQR: −56.73 to +16.63) vs −US$10.77 (IQR:−77.23 to +11.60), p=0.556).

The proportion of participants living in poverty increased from 41.6% (166/399) to 57.7% (211/366) over this time (p<0.001), but the proportion of participants reporting that they were the highest earner in the household did not change (pre-illness: 57.0% (231/405); TB-treatment end 54.6% (221/405); 1-year post treatment completion 60.6% (223/368)).

#### Healthcare costs

Direct healthcare costs in the year after TB-treatment completion were limited. Among those contributing any follow-up data, two-thirds of participants (66.8%, 254/380) had ≥1 outpatient visit, including 264 planned and 173 unscheduled visits, and 6.3% (24/380) had ≥1 inpatient admission. The majority of both outpatient visits (95.0%, 415/437) and admissions (88.9%, 24/27) occurred within the public sector. The majority of planned visits (87.1%, 230/264) were for appointments at ART clinics.

The median direct cost of an outpatient visit, including both planned and unscheduled visits, was US$1.05 (IQR: 0.14–2.09), including expenditure for clinic fees and medications, and travel, accommodation, food and phone time for patients and guardians. The median time taken for any outpatient visit was 3 hours (IQR: 2–4), and loss of income was reported for 53.1% (232/437) of these visits, with average income time loss of 1 hour (IQR: 0–1) only. Guardians attended with study participants for a minority (8.2%, 36/437) of outpatient visits, and on these occasions rarely reported income time lost (5.6%, 2/36).

The median duration of the 27 inpatient admissions was four nights (IQR: 2–19), with median direct costs of US$19.62 (IQR 13.30–61.91). Lost income was reported by participants for under a third of admissions (29.6%, 8/27), and although participants were accompanied by a guardian for the majority of these admissions (88.9%, 24/27) only one guardian reported lost income.

The proportion of participants attending ≥1 outpatient visit was similar in the lowest two, and top three socioeconomic quintiles (66.2% (73/107) vs 66.8% (177/265), p=0.790), and median per-visit costs were similar between socioeconomic groups (US$0.84 vs US$1.12, p=0.578). A higher proportion of participants from the lowest two socioeconomic quintiles required hospital admission compared with the top three quintiles (10.3% (11/107) vs 4.9% (13/265), p=0.056) but median per-admission costs were similar between groups (US$19.27 vs US$19.97, p=0.750).

### Impact of economic morbidity, on patients and households

Interruption of a child’s schooling due to the financial impact of illness was reported by 17.0% (69/405) and 9.5% (35/368) of TB-affected households in the years prior to and after TB-treatment completion, respectively. School interruptions were more common in the lowest two, compared with the top three, socioeconomic quintiles (32.1% (34/106) vs 17.7% (45/255), p=0.003).

Over a third of participants (37.0%, 150/405) reported that TB had had a severe financial impact on their household, graded ≥4/5 on a Likert scale, at TB-treatment completion. This proportion was 16.9% (62/368) 1 year later. Self-reported severe financial impact was more common in the lower socioeconomic strata (58.5% (62/106) vs 37.4% (96/257), p<0.001).

Almost three-quarters of participants reported dissaving by the point of TB-treatment completion (73.6%, 298/405), and half reported dissaving in the following year (50.5%, 186/368), at values of 54.9% (IQR:24.3%–146.4%) and 53.2% (IQR:19.0%–125.7%) of the baseline monthly income prior to TB illness respectively. Over a quarter (26.7%, 27/101) of those with no dissaving during TB disease and treatment did go on to report dissaving in the year after treatment completion. Dissaving was more common in lower SES quintiles, but with lower absolute and relative values (table 2).

**Table 2:** Prevalence and value of dissaving in the years prior to and after TB-treatment completion, stratified by wealth quintiles*

Time period	Malawi urban wealth quintile (Q1–Q5)†					All participants
	Q1 (n=56) Wealthiest	Q2 (n=114)	Q3 (n=95)	Q4 (n=85)	Q5 (n=22) Poorest	
**Prior to TB illness (n=372)**						
Predisease monthly income (US$)	108.30 (0–270.76)	83.03 (39.71–287.73)	63.18 (25.27–158.84)	61.37 (25.27–111.91)	41.52 (10.83–121.30)	72.20 (25.27–173.29)
**During TB illness and treatment (n=372)**						
Proportion incurring any dissaving	28/56 (50.0%)	83/114 (72.8%)	75/95 (79.0%)	68/85 (80.0%)	20/22 (90.9%)	274/372 (73.7%)
Value of dissaving, if experienced (US$)‡	166.9 (94.58–423.50)	69.54 (34.77–173.85)	38.94 (15.30–115.44)	27.82 (16.69–80.95)	33.38 (13.91–100.39)	55.63 (20.86–139.08)
% of predisease monthly income (median, IQR)	89.2% (34.1–266.0)	58.2% (24.1–152.3)	53.9% (20.7–111.2)	41.6% (23.4–104.6)	44.1% (35.0–173.4)	54.9% (24.3–146.4)
**Year after TB-treatment completion (n=360)**						
Proportion incurring any dissaving	12/55 (21.8%)	48/107 (44.9%)	61/92 (66.3%)	50/84 (59.5%)	13/22 (59.1%)	184/360 (51.1%)
Value of dissaving, if experienced (US$)‡	167.6 (59.36–272.35)	69.83 (24.1–152.3)	39.11 (20.95–92.18)	37.71 (13.97–69.83)	20.95 (11.17–31.42)	41.9 (20.95–94.97)
% of predisease monthly income (median, IQR)	112.5% (49.0–232.1)	77.6% (19.0–198.7)	59.3% (24.2–89.3)	41.9% (13.6–120.7)	29% (15.0–58.0)	53.2% (19.0–125.7)

*Values given for those who experienced dissaving, only.

†Baseline SES missing for 33 participants—data included for 372 participants only.

‡Standardisation into US$ using exchange rates at midpoints of first and last study visits.

SES, socioeconomic situation; TB, tuberculosis.

**Figure 4:**
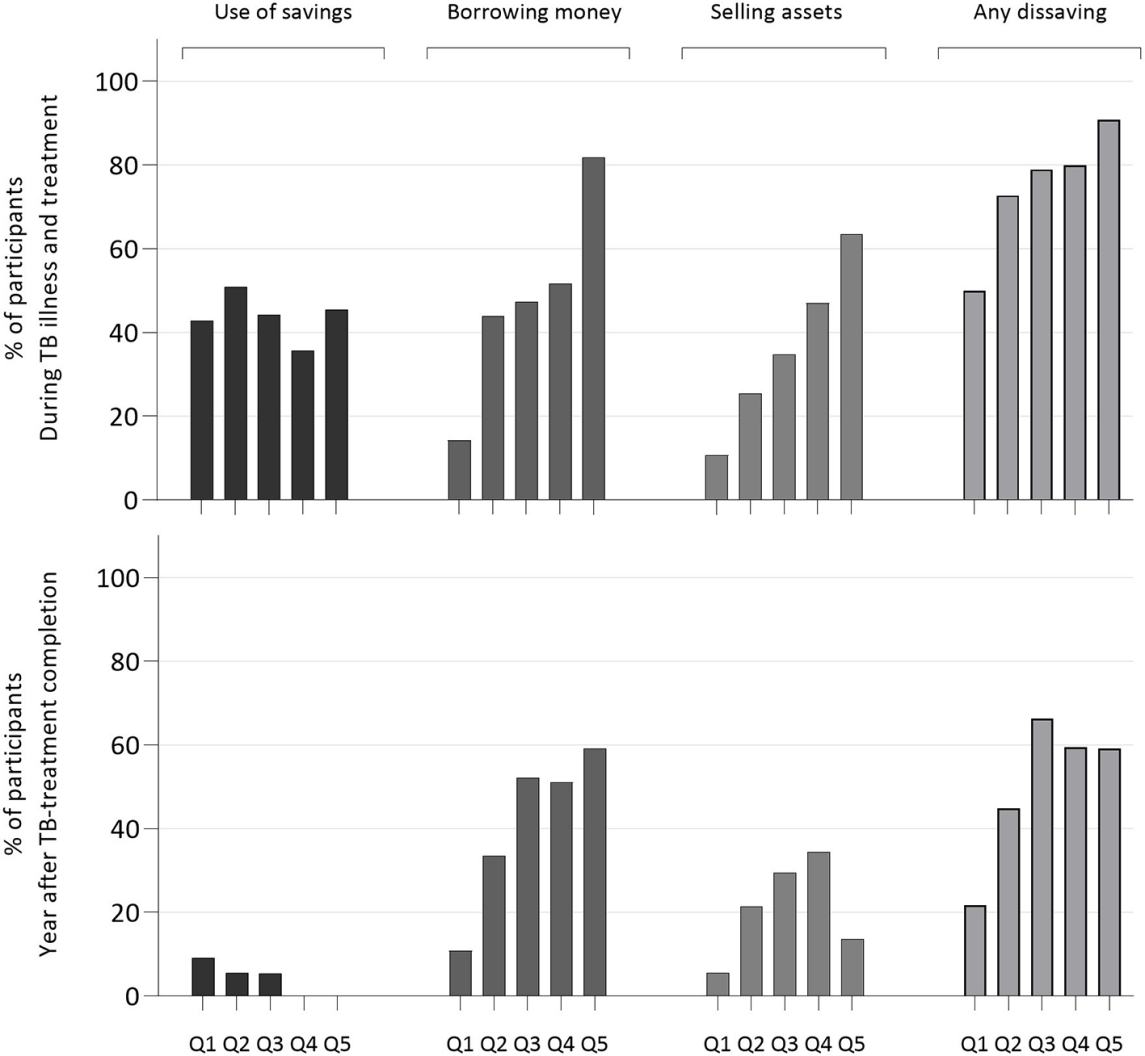
Proportion of participants incurring patterns of dissaving, during TB illness and treatment (n=372), and in the year after TB-treatment completion (n=360), stratified by urban wealth quintile (Q1–Q5)*. Q1 – Q5: richest to poorest urban wealth quintiles, calculated at TB treatment completion using the MalawiEquityTool 2012. *Wealth quintile data missing for n=33 participants.TB, tuberculosis.

Patterns of dissaving varied by SES group, and over time (figure 4). All wealth strata used savings during the period of TB illness and treatment, but only the wealthiest quintiles used savings during the subsequent year. Borrowing money was the most common among lower socioeconomic strata, and over half of those in the lowest quintiles reported borrowing money in the year after TB-treatment completion. The most common sources of borrowed money were friends (44.8%, 172/384), family (26.8%, 103/384) and the black market (10.2%, 39/348)—use of the latter increased from 10.7% (19/177) in the first year to 17.1% (25/246) in the second year. The sale of assets to cover costs due to illness during the period of TB illness and treatment was also more common in poorer groups. The most common items sold were household items (35.0%, 134/383) and mobile phones (10.7%, 41/383). Potentially income-generating assets sold included land (1.3%, 5/383), livestock (3.7%, 14/383) and means of transport (4.7%, 18/383).

### Relationship with TB retreatment

TB retreatment was initiated in 15/405 (3.7%) of participants, of whom five died, one relocated, and nine completed study follow-up. Socioeconomic outcomes were poor among those who survived and completed follow-up: by 1 year after TB-treatment completion, a higher proportion of those receiving retreatment had lost work (33.3% (3/9) vs 20.3% (71/349), p=0.342), experienced dissaving (100% (9/9) vs 79.4% (285/359), p=0.128), or reported a severe financial impact from TB disease (33.3% (3/9) vs 16.4% (59/359), p=0.181), compared with those who did not receive retreatment. However, none of these differences were statistically significant.

### Relationship between physical and economic morbidity

Almost one-third of participants had abnormal spirometry (30.7%, 103/336) or regular respiratory symptoms (30.7%, 113/368) 1 year after TB-treatment completion. Those with abnormal spirometry were more likely to have lost work in the period from TB-illness onset, compared with those with normal spirometry (OR 1.87, 95% CI 1.02 to 3.41). Those with residual symptoms were more likely to report that TB had had a severe financial impact on the household, compared with those without symptoms (2.02, 95% CI 1.10 to 3.68). Those with chest symptoms limiting their ability to keep up with peers (17.4%, 64/368), interfering with work (12.2%, 45/368) or limiting activities (4.4%, 16/368) at 1 year were significantly more likely to have experienced both of these outcomes. No significant association was observed between these measures of physical morbidity and the use of dissaving or change in income from pre illness to 1-year post TB-treatment completion (table 3).

**Table 3:** Adjusted ORs for associations between respiratory morbidity at 1-year post treatment completion, and economic outcomes over the study period, in multivariate analyses controlling for patient characteristics (age, gender, HIV status, TB microbiology, and educational level) at TB treatment completion

Physical morbidity	Economic outcome from prior to TB illness, to 1-year post-treatment completion			
	Loss of work (OR, 95% CI)	Use of dissaving (OR, 95% CI)	Income difference (β-coefficient, 95% CI)	Self reported severe financial impact of TB at 1 year (OR, 95% CI)
Abnormal spirometry at 1 year No Yes	1.0 1.87 (1.02 to 3.41)	1.0 1.31 (0.71 to 2.43)	11.64 (–50.80 to 74.09)	1.0 1.85 (0.96 to 3.58)
	p=0.042	p=0.393	p=0.714	p=0.066
Respiratory symptoms at 1 year No Yes	1.0 1.26 (0.72 to 2.20)	1.0 0.79 (0.45 to 1.37)	−6.34 (–63.01 to 50.33)	1.0 2.02 (1.10 to 3.68)
	p=0.412	p=0.399	p=0.826	p=0.022
Difficulty keeping up with peers when walking at 1 year No Yes	1.0 2.39 (1.27 to 4.49)	1.0 0.61 (0.32 to 1.18)	7.26 (–62.80 to 77.31)	1.0 2.04 (1.03 to 4.04)
	p=0.007	p=0.145	p=0.839	p=0.041
Chest symptoms interfering with/stopping work at 1 year No Yes	1.0 4.13 (2.06 to 8.28)	1.0 1.04 (0.45 to 2.39)	32.73 (–47.09 to 112.56)	1.0 4.24 (2.08 to 8.66)
	p<0.001	p=0.934	p=0.421	p<0.001
Chest symptoms limiting most/all daily activities at 1 year No Yes	1.0 9.35 (3.02 to 28.95)	1.0 0.56 (0.17 to 1.85)	12.51 (–118/28 to 143.29)	1.0 11.82 (3.74 to 37.33)
	p<0.001	p=0.340	p=0.851	p<0.001

Income difference: US$ standardised change in monthly income from prior to TB disease, to 1-year post-TB-treatment completion.

Use of dissaving: any use of savings, borrowing of money or selling of assets from the onset of TB disease to 1-year post TB-treatment completion.

Loss of work: no longer working, having been in work (employed or self-employed) prior to TB disease.

TB, tuberculosis.

### Illness narratives

The persistent socioeconomic impact of TB disease was evident in the illness narrative data (table 4). The shift to a lower standard of living after TB disease was raised as a barrier to ongoing health and well-being. The need for further dissaving and withdrawal of children from school to release extra funds were highlighted as areas of concern.

**Table 4:** Quotes from in-depth interviews with TB survivors

Theme	Quote
**Impact of TB-related financial hardship on participants and households**	
Reduced standard of living	‘Since the time I was diagnosed with TB until now, I am staying in a bad looking house, with bad sleeping environment along with bad food’ (female participant, 39 yrs ‘As [name of participant] hasn’t been able to find work since he completed his treatment, the family had to move up the hill, where housing is cheaper. They also had to sell most of their furniture. There was only a mat on the floor, a little stool and a couple of mugs for a family of four…Another interesting observation we made relates to his relocation. Moving to cheaper accommodation on top of the hill means that he leaves the house less, as physical exercise remains a big challenge for him. This in turn limits his occupational activities and affects his health seeking behavior’ (Research Assistant, relating to male participant, 32 yrs)
Anxiety around debt and financial insecurity	‘I never used to have financial difficulties. Now, my business is just so small with borrowed capital and the creditors keep coming to my house, saying they want their money. If I fail to pay, there will be bigger interest. I have been in debts ever since I completed the treatment’(female participant, 29 yrs) ‘Now, I am not having anything to eat and sometimes I don’t have money to pay for rent. For example, I haven’t paid rent yet. In the past, before I became sick, I could pay rent in advance’ (male participant, 34 yrs)
	‘I have been facing financial challenges, lack of food and so on. In 2016, my girl failed to write her form two exams, due to lack of schooling that I couldn’t pay for. So, I have faced so many difficulties from the time that I was diagnosed with TB until now’ (female participant, 39 yrs) ‘I used to sell our house equipment to sustain my family. So, we sold our TV and some small items. Others who could help us were living far from us and they can’t just be helping you every day. Our children stopped going to school, so we had to sell whatever household equipment we had to sustain our living’ (male participant, 32 yrs)
Dependence on others	‘As of now, I have difficulties to get food, but I do try my level best to hunt for money to buy the food. My family supports me since I’ve completed treatment. Whenever I say that I don’t have money to pay rental expenses and to buy food, they do send me the money’ (female participant, 27 yrs)
Loss of social standing	‘While I was sick and during the time when I completed my treatment people were not respecting me, but people were respecting me before I became unwell with TB. I think this is because I lost my income, and my family helps me. When you have money, people tend to respect you’ (male participant, 34 yrs) ‘He feels, once you have money, you have so much power and you can tell your employees what to do. In his case, his employees overtook power while he got sick, which still affects him’ (Research Assistant, relating to a male participant, 34 yrs) When asked about how the income loss affects life: ‘It has affected me a lot. I just feel depressed and sometimes I wonder if I am the same person’. (male participant, 18 yrs)
**Barriers to income recovery**	
Ongoing physical morbidity	‘There are so many problems, I am facing these days because everything needs money… I still need to work, so I do some piece work, whether it means that I am still feeling pain, but I do work in order to get money to help myself…. The most important thing is to get money, so if you don’t work then you have to do business in order to maintain your health and to fulfil your needs’ (male participant, 37 yrs)
Stigma	‘My boss said that I should wait at home during treatment…My boss accepted my TB diagnosis, but she didn’t want me back after I completed’ (female participant, 42 yrs) ‘I went back to my work, but my boss discriminated against me and he told me that he wouldn’t be helping me anymore financially, so I am just staying here at home’ (male participant, 32 yrs) ‘They [colleagues] would be surprised to see my work performance, which was dropping as I could sometimes work well one day and sometimes, I could not work well …they were saying that it was AIDS’ (male participant, 18 yrs) ‘The wife of the participant told us that she sells food items in front of the house and noted that some people don’t want to buy from her, because they know that her husband is sick’ (Research Assistant, relating to male participant, 32 yrs)
Loss of social and work relationships	‘Our customers really had forgotten us, so I think it will take time for me to grow the business again’ (male participant, 33 yrs)
Lack of capital for reinvestment	‘My life has changed now …I have little capital … I don’t do hard work now, so my employees help me do business. My business isn’t the way it was before, because some of my business centers are closed now, I stopped selling Irish potatoes, I closed my take-away shops and I only have one bench of chips’ (male participant, 48 yrs) ‘The TB symptoms affected my business so much, to the extent that it went down up to date and it’s not at all growing, though I was cured…My husband cannot even afford to give me MK 20,000.00 to start up a new business. I went to borrow money from someone on interest, but I haven’t paid the person back. The capital you have determines what kind of business one engages in. So, instead of starting up a business with the little money borrowed, you start buying maize to feed children at home’ (female participant, 39 yrs)

TB, tuberculosis.

Anxiety around loss of financial security, debt and the challenge of ongoing dependence on family and friends for support emerged as strong themes. A gendered response was seen with a perceived loss of social standing due to this dependence particularly common and problematic for men.

Reasons for limited recovery of income and work were explored. Ongoing physical morbidity was noted as a challenge to patients’ livelihoods, however, participants largely continued to work despite residual symptoms in order to maintain income. Stigma was widely experienced and resulted in delayed return to work, or loss of work for those previously employed, with discrimination from colleagues often rooted in the perception that TB and HIV disease are linked. Loss of business infrastructure and the lack of capital to rebuild and reinvest was highlighted. Participants reported challenges in rebuilding business relationships which had been lost after a prolonged absence during the illness period, including those with employers, employees and customers.

## Discussion

In this study, we explore the long-term socioeconomic consequences of TB disease after TB-treatment completion. Our data show that the substantial financial insult experienced during TB illness extends to 12 months post-treatment completion. Economic recovery in the year after TB-treatment was slow and incomplete, with many patients continuing to experience income loss and reduced work. Persistent dissaving was widely observed and suggests increasing financial vulnerability. A substantial minority of patients experienced ongoing respiratory morbidity after treatment completion, and this was significantly associated with economic morbidity. Additional barriers to recovery after TB-treatment completion included ongoing financial insecurity from initial TB disease, reduced social capital and TB-related stigma.

Even in settings where TB services are free of charge within the public sector, the financial impact of TB disease is marked: a 2014 systematic review of 49 studies found that on average patients lost the equivalent of 58% (range 5%–306%) of annual individual income and 39% (range 4%–148%) of annual household income in direct and indirect costs during TB illness and treatment, with half of all costs incurred prior to treatment initiation.^3^ Those incurring ‘catastrophic’ costs (≥20% of annual household income) have been shown to have higher odds of adverse TB-treatment outcomes (death, treatment failure or recurrence).^4^

Our results support these findings of a major initial TB-related financial insult: during TB disease and treatment employment decreased, average patient incomes fell, and three-quarters of the cohort incurred dissaving. This pattern was seen across socioeconomic and employment groups, and by the end of TB-treatment the majority of TB survivors were living in poverty.

However, our data also show that this impact is sustained, even after TB-treatment completion. Although the proportion of participants working increased in the year after TB-treatment completion, it did not return to baseline: 1 year after TB treatment, almost one-third of patients were unemployed, with standardised individual incomes lower than prior to illness. Self-employed individuals appeared particularly vulnerable, with large drops in income experienced by both those who were in work through their disease, as well as those who stopped working.

Post-TB physical morbidity was associated with limited recovery: abnormal spirometry, ongoing respiratory symptoms and chest symptoms limiting activity at 1 year were strongly associated with loss of work and perceived financial severity of the TB illness episode in multivariate analyses. Post-TB physical morbidity is increasingly recognised as a key component of the overall number of disability-adjusted and quality-adjusted life-years lost in relation to TB disease,^24^ and our findings suggest that its impact on long-term productivity and financial vulnerability should also be considered.^25^ Recurrent TB disease may also be detrimental to this group: socioeconomic outcomes among those receiving TB retreatment were poor in this study, but our ability to explore this finding was limited by the low numbers of retreatment patients identified, and further work is needed in this area.

In-depth interviews highlighted the loss of business assets during disease, with limited access to capital to rebuild after treatment completion as a major barrier to recovery. The challenge of rebuilding relationships with employers, employees and clients was emphasised, and attributed to a prolonged period of absence during TB illness and treatment, as well as loss of social standing due to impoverishment, disability, and the direct consequences of TB-related and HIV-related stigma. The impact of TB-related stigma on patients’ emotional well-being has been documented elsewhere,^26^ but our data suggest that this also has socioeconomic repercussions.

Rather than promptly recovering, our data suggest that many TB survivors are at risk of further financial and psychological decline after TB-treatment completion. Dissaving is a coping mechanism for catastrophic costs,^2^ and has been widely observed in TB-affected households during TB illness and treatment.^27^ However, in this study, dissaving was observed in half of the cohort in the year after TB-treatment completion, including several households who had resisted dissaving during initial disease and treatment itself. Concern about dissaving was widely reported in the qualitative data. These findings suggest that even after TB-treatment completion, households continue to deplete their reserves or enter into further debt as they struggle to cover costs or seek to rebuild their lives and livelihoods.

Of particular concern, both the use of savings and the sale of assets declined in the post-treatment period, particularly among low socioeconomic strata, perhaps reflecting depletion of these resources. Instead, borrowing of money remained widespread with increasing use of the black market for loans, perhaps reflecting the exhaustion of more ‘benign’ sources of loans such as friends and family.

Interruption of children’s education continued in 10% of households in the year after TB-treatment completion, and a high burden of anxiety related to financial insecurity, lower standard of living, and school interruptions was observed among TB survivors. Men voiced concern around loss of social standing, which is consistent with previous work describing high societal pressures on men to be effective providers, regardless of the difficulties of their circumstances.^28^ Taken together, our qualitative and quantitative data suggest that TB disease may push patients into an ongoing cycle of poverty, with many patients become increasingly financially vulnerable after TB-treatment completion, rather than experiencing financial recovery.

This study was performed at a single site and work from other resource-limited settings is needed to confirm findings. In the absence of a control group we cannot exclude the possibility that changes observed were related to general changes in the economy, although this is unlikely as unemployment within Malawi was falling over the study period, changes in income persisted despite time-dependant standardisation into USD, and findings were consistent across qualitative and quantitative data.^23^ Data on incomes and occupation prior to TB-illness onset, and healthcare costs between study visits were collected retrospectively, with some risk of recall bias. Our analyses used individual rather than household-level income data, perhaps leading us to underestimate participants’ access to financial resources. The financial compensation provided for study participation may have acted as an additional source of income, cushioning participants from the full financial hardship which may have been experienced under routine conditions.

Strengths of this study include its novel focus on patients’ lives and livelihoods after TB-treatment completion, and use of mixed methods to understand participant perspectives and experiences. Qualitative data were collected to saturation, and the economic tools used were derived from validated sources. The study was conducted in an unselected population, with broad inclusion criteria, and minimal loss to follow-up, allowing broad generalisability.

Our findings have several key implications for TB research, policy and programmes. We recommend that studies investigating costs associated with TB disease should measure economic outcomes beyond TB-treatment completion. Recent data suggest that mortality rates among TB survivors are higher than that of TB-naïve individuals, and the extent to which the socioeconomic impact of TB disease contributes to this requires further investigation. Our findings suggest that interventions to protect livelihoods and prevent dissaving during disease may be crucial to the long-term well-being of TB-affected households. Microloans and training programmes to assist TB survivors to rebuild their livelihoods after treatment completion must be explored. These interventions should be codeveloped with TB-affected communities, and must be accompanied by community-level education programmes to address TB-related stigma. Ultimately, a renewed focus on physical, psychological and socioeconomic well-being after TB-treatment completion is needed if we are to improve the long-term outcomes of TB survivors.
