# Supplementary material for: The long term effect of pulmonary tuberculosis on income and employment in a low income, urban setting
Source: Thorax. 2020 Dec 18;76(4):387–95. doi: 10.1136/thoraxjnl-2020-215338 (PMC7982936; doi:10.1136/thoraxjnl-2020-215338)
Supplement: Supplementary data [file thoraxjnl-2020-215338supp001.pdf]

Supplementary materials

Title: Beyond TB treatment completion: The lasting impact of pulmonary TB on incomes and livelihoods in an urban setting in sub-Saharan Africa

Authors: Jamilah Meghji, Stefanie Gregorius, Jason Madan, Rachael Thomson, Fatima Chitimbe, Jamie Rylance, Ndaziona PK Banda, Stephen B Gordon, Elizabeth L Corbett, Kevin Mortimer, S Bertel Squire

Meghji J, *et al. Thorax* 2021; 76:387–395. doi: 10.1136/thoraxjnl-2020-215338

S1 Appendix: US Dollar standardisation of income data

Table 1: Standardisation of income data using USD exchange rates, over duration of study

Data collection period	Date range for data collection	Mid-point	MK:$US exchange rate, at mid-point
Prior to TB-disease	Feb 15 – May 16	16th October 2015	553.93
TB treatment completion	16th Feb 16 – 16th May 17	16th October 2016	718.75
12-months after treatment completion	6th Feb 17 – 26th April 18	16th October 2017	715.71

Meghji J, *et al. Thorax* 2021; 76:387–395. doi: 10.1136/thoraxjnl-2020-215338

S2 Appendix: In-depth interview topic guide: Investigating the lived experience of respiratory disability following pulmonary TB (PTB) amongst adults in Blantyre, Malawi

1. Introduction to interview

You are being asked to take part in a research study on the experiences of people with lung health problems and how these problems affect their lives and how they deal with their health. Before you decide whether or not you wish to take part, it is important for you to understand why the research is being done and what will be involved. Please ask us if anything is unclear or you would like more information.

2. Participant and interview characteristics

Interview ID	
Date of interview	
Location of interview	
Interviewer	
Gender of participant	
Age of participant	
Marital status of participant	
Occupation of participant	
Educational level participant	

3. Health over the life course

Now, we would like to learn in more detail about how your lung health (pulmonary impairment) after TB treatment affects different aspects of your daily life, including your work, family and social life, as well as your health in general.

3.1. Life before TB

Can you please tell me about your life before you became unwell with symptoms of TB, and before you were diagnosed / started treatment?

- Health status
  - What did you understand about TB, before you became unwell?
  - How was your health, before you became unwell with TB? Did you have any other health problems?
  - What did you know about TB before your symptoms/diagnosis?
- Livelihood
  - What work were you doing before you became unwell?
  - What were the major source(s) of income, for you and your household? PROMPTS: education, multiple/single occupations, regular/irregular income
- Household activities
  - What were your main household activities and how could you perform them? PROMPTS: physically challenging or easy; emotionally challenging or easy; time-consuming or not
- Role within family

- What were your role(s)/duty(ies) within the family and how were you treated within this role? PROMPTS: breadwinner, care taker, parent, child etc.; level of respect within family; role in decision making process

- Role within the community
  - What was your role within the community and how did other people in the community treat you? PROMPTS: affiliations and respective functions, level of respect
  - What did you do in your spare time and with whom
- Other role(s)

3.2. Life during TB onset, diagnosis and treatment

NB for interviewer: This section covers three time periods:

A. When unwell prior to diagnosis
B. At the time of diagnosis itself
C. Whilst on treatment

Experiences may have been different for patients in all three periods. Therefore, please ask about all of these periods (2A, 2B, and 2C) in the following this section.

We would like to know what happened and how life changed when you first experienced TB symptoms, and when you were diagnosed and treated for TB.

Prior to diagnosis

- Health care seeking
  - Can you please describe how your health changed when your first experienced symptoms?
  - How did you manage your symptoms?
  - How did you decide to seek care?
    - Who was involved in the decision?
  - Where did you decide to seek care and why did you choose this place?
    - Did you go to single/multiple place(s)
    - What costs were involved?
    - How far was it to get there?
    - How would you assess the availability of health care facilities?
    - How would you assess the quality of the health care facility/ies you used?
  - What happened when you went to seek care?
  - How were you treated by the staff?
    - How was the staff's attitudes and behaviour towards you?
    - What kind of information did you receive about diagnosis and management and how do you assess this information
- Livelihood
  - How did the TB symptoms affect your occupational/educational activities?
    - Did you have to stop work/school?
    - How did it impact your performance?
    - How did it impact your income?
    - Did you tell your boss/colleagues/peers? If yes, how did they react? PROMPT: Level of support/discrimination
- Household activities

- How did the TB symptoms affect your activities in your household? PROMPT: Level of difficulty to perform, changes in activities
- Role within family
    - Did you tell your family about symptoms?
        - If yes, who did you tell and why? How did they react and how did they treat you?
        - If no, why did you not tell them?
    - How has your role in your family changed when you first experienced symptoms?

- Role within the community
    - What is the perception about TB in your community?
    - Did you tell in your community/social networks about your symptoms?
        - If yes, who did you tell and why? How did they react and how did they treat you?
        - If no, why did you not tell them?

TB Diagnosis

- Experience of TB diagnosis
    - How did you feel when you received your diagnosis?
    - What happened when you receive the diagnosis?
        - How did the staff treat you?
        - How do you assess the information you received?
    - Did you tell others about your diagnosis? Who did you tell & why?
    - Did you seek health care from somewhere else when you were diagnosed?
        - If yes, from where and why?
- Livelihood
    - How did the diagnosis affect your occupational/educational activities?
        - Did you have to stop work/school?
        - How did it impact your performance?
        - How did it impact your income?
        - Did you tell your boss/colleagues/peers? If yes, how did they react? PROMPT: Level of support/discrimination
- Household activities
    - How did the diagnosis affect your activities in your household? PROMPT: Level of difficulty to perform, changes in activities
- Role within family
    - Did you tell your family about your diagnosis?
        - If yes, who did you tell and why? How did they react and how did they treat you?
        - If no, why did you not tell them?
    - How has your role in your family changed when you received the diagnosis?
- Role within the community
    - Did you tell in your community/social networks about the diagnosis?
        - If yes, who did you tell and why? How did they react and how did they treat you?
        - If no, why did you not tell them?

TB treatment

- Experience of TB treatment
    - What kind of treatment did you receive?

        - What influenced your treatment and adherence to treatment?
    - How did you find the treatment?
    - How did you get your treatment each month – how did you find this process?
    - Did you experience any other health care needs during your TB treatment?
        - If yes, what were they and how did you take care of them?
- General health care experience
    - What was good about your overall health care experience with regard to symptoms, diagnosis and treatment?
    - What was bad about your overall health care experience with regard to symptoms, diagnosis and treatment?
- Livelihood
    - How did the TB treatment affect your occupational/educational activities?
        - Did you have to stop work/school?
        - How did it impact your performance?
        - How did it impact your income?
        - Did you tell your boss/colleagues/peers? If yes, how did they react? PROMPT: Level of support/discrimination
- Household activities
    - How did TB treatment affect your activities in your household? PROMPT: Level of difficulty to perform, changes in activities
- Role within family
    - How has your role in your family changed since on treatment?
- Role within the community
    - Did you tell in your community/social networks about treatment?
        - If yes, who did you tell and why? How did they react and how did they treat you?
        - If no, why did you not tell them?
- Other
    - E.g: What kind of support did you need in the above aspects of your life and how did you go about receiving this support

3.3. Life after TB treatment

Can you please tell me what and how life changed since you completed treatment?

- Health status, health care seeking behaviour and health care experience?
    - Can you please describe your health since you've completed treatment?
    - Do you still have any problems with your health now?
        - If yes, what are they and how do you take care of them?
        - Who is involved in making decisions about seeking health care?
        - What influences your decisions and why? PROMPTS: Cost, distance, availability, quality
    - Where do you seek care and why? PROMPTS: single/multiple place(s)
    - How has your TB treatment experience influenced your health care seeking behaviour?
- Livelihood
    - How have your occupational/educational activities changed since you've completed treatment?
        - What kind of work/education are you currently performing?

        - Since treatment completion how does your health affect the type of work/education you are doing?
        - How does your health influence your performance?
        - How do your boss/colleagues/peers treat you since you completed treatment? PROMPT: Knowedge of treatment, Level of support/discrimination
- Household activities
    - How does the completion of treatment affect your activities in your household? PROMPT: Ability to perform, changes in activities
- Role within family
    - What changed in your family life since you've completed treatment?
    - What role does your family play in your life since you've been completed treatment?
- Role within the community
    - What has changed in your community life since you've completed treatment?
    - What role does your community/social network play in your life since you've completed treatment?

3.4. The future

Can you tell me what your hope for the future is in terms of health, livelihood, community life?

- What would help you achieve these hopes?
- What do you think limits you in achieving these hopes?

S3 Appendix: Comparison of participants who did / did not complete study follow-up

Table 2: Baseline characteristics of participants who did / did not complete study follow up

Participant characteristic	Completed 12m follow-up (n=368)	Lost to follow-up (n=37)	p-value∞
Age (yrs) (median, IQR)	35 (28.5 – 41)	34 (28 – 39)	0.632
Male sex (n, %)	249 (67.7%)	26 (70.3%)	0.746
Positive TB microbiology* at diagnosis (n, %)	287 (78.0%)	26 (70.3%)	0.285
HIV-infected at TB-treatment completion (n=403)^	225 (61.3%)	19 (52.8%)	0.318
Antiretroviral treatment use at TB-treatment completion (n, %) (n=244)	206(91.6%)	18 (94.7%)	0.627
CD4 if HIV-positive at TB-treatment completion (cells/µL) (n=242)	244 (137-398)	113 (62 – 197)	0.007
Maximum education level > primary school (n, %)	225 (61.1%)	26 (70.3%)	0.276
Urban SES quintile (n=372)†			
- Poorest	22 (6.1%)	0 (0%)	0.376
- 2nd poorest	84 (23.3%)	1 (8.3%)	
- Middle	92 (25.6%)	3 (25.0%)	
- 2nd most wealthy	107 (29.7%)	7 (58.3%)	
- Most wealthy	55 (15.3%)	1 (8.3%)	

*Microbiology positive if smear, culture or Xpert MTB/RIF positive;

^HIV status missing for 2 study participants;

† Urban household wealth quintiles calculated from household characteristic and asset data, using a tool validated by the Malawi Malaria Indicator Survey 2012 (Equity measurement Toolkit; Social Franchising Metrics Working Group). Missing data for n=33 participants;

∞Chi^2 or Fisher's exact test for categorical variables, Wilcoxen rank sum for continuous variables

Meghji J, *et al. Thorax* 2021; 76:387–395. doi: 10.1136/thoraxjnl-2020-215338
